# Innovative Assessments Help Elucidate Sustained Improvements in Fitness and Metabolic Health in Obese Children

**DOI:** 10.21767/2572-5394.100020

**Published:** 2016-11-16

**Authors:** Cassandra M Vanderwall, R Randall Clark, Jens C Eickhoff, Aaron L Carrel

**Affiliations:** 1 UW Health-University of Wisconsin Hospitals and Clinics Pediatric Fitness Clinic, USA; 2 Department of Pediatrics, University of Wisconsin School of Medicine and Public Health, USA

**Keywords:** Childhood obesity, Cardiovascular fitness, Body composition, Body mass index (BMI), Multidisciplinary clinic, Body fat percentage, Weight management

## Abstract

**Objective:**

Treatment of childhood obesity is a medical challenge and limited data are available describing successful long term interventions. This study presents a multi-disciplinary intervention that resulted in sustained physiological improvement over a one-year period.

**Methods:**

The criterion outcome variables include cardiovascular fitness (CVF) measured by a population-specific treadmill test to predict maximal oxygen uptake (predicted VO_2_ max) and the body composition (BC) variables of fat mass, non-bone lean mass and percent body fat from whole body dual energy x-ray absorptiometry (DXA) scans. Subjects were overweight and obese children (N=79) evaluated at baseline, 6 and 12 months at a University Hospital-based pediatric fitness clinic.

**Results:**

Statistically significant improvements in non-bone lean body mass (+4.24 kg ± 5.0, p<0.0001) and predicted VO_2_ max (+0.14L/min ± 0.10, p<0.0001) were seen at 6 months. These significant improvements were sustained over 12 months: body fat percentage (−2.28 ± 3.49, p<0.0001), lean mass (+6.0 kg ± 4.0, p<0.0001) and predicted VO_2_ max (+0.22 L/min ± 0.19, p<0.0001). These results were observed despite increases in weight and body mass index (BMI) at 6-months (weight: +6.6 kg ± 6.93, p<0.0001; BMI: +0.37 ± 1.21, p=0.47) and 12-months (weight: +6.3 kg ± 5.8, p<0.0001; BMI: +0.91 ± 2.06, p=0.0002).

**Conclusion:**

These results reflect the sustained effect of a multidisciplinary approach, and the value of using valid and reliable assessment methods to measure sustained physiological changes in a sample of 79 overweight and obese children.

## Introduction

Childhood obesity is associated with both short- and long-term morbidities including increased cardiovascular risk factors, insulin resistance and type 2 diabetes, hypertension, dyslipidemia, asthma, obstructive sleep apnea, psychosocial problems, decreased quality of life, and increased likelihood of becoming obese adults [1-12]. Poor cardiovascular fitness (CVF) is an independent risk factor for insulin resistance9 and a strong predictor of morbidity [13-20]. Therefore, assessing CVF and addressing habits that increase risk for obesity early is a key strategy for preventative and therapeutic intervention of childhood obesity [21-25].

Collectively, the aforementioned morbidities associated with childhood obesity highlight the need for individualized assessment and the importance of early intervention to prevent obesity and improve long-term health outcomes. Primary care providers play a pivotal role in the process of preventing, identifying and treating childhood obesity and associated comorbidities [26-31]. However, many providers in the primary care setting feel unable to thoroughly assess the needs and accomplish lifestyle change in these complex patients and families [27-29]. There is a need for specialty clinics that are able to implement evidence-based obesity assessment, prevention and management strategies, such as those described by the American Academy of Pediatrics (AAP), the Alliance for a Healthier Generation, and the Academy of Nutrition and Dietetics (AND) [2,27-35]. At present, long-term outcomes of childhood obesity remain mixed and many providers report frustration with a paucity of long-term data [35]. This may be because providers are unable, or unaware, of useful methods for measuring sustained improvements in fitness in obese children. This cross-sectional study of subjects who completed a one-year clinical intervention aims to augment the literature and provide evidence for alternative methods for measuring and evaluating progress in a pediatric population.

## Materials and methods

### Subjects

Subjects were 79 children (49% male) with median age of 11 years (4 to 18 years) who were completed a 12-month intervention in the Pediatric Fitness Clinic- a comprehensive weight management program. The sample was predominantly English-speaking (76%) and half (51%) utilized private insurance versus public.

### Design and setting

The multidisciplinary weight management intervention included a medical evaluation, individualized consultation from a registered dietitian (RD) and exercise physiologist (EP), and anthropometric, cardiovascular fitness (CVF), dietary and body composition (BC) assessments. The intervention was individualized to the patient and family, and included an initial visit with the entire clinical team, followed by medical reassessment by the physician and follow-up visits with the RD and EP every four weeks for one year. The purpose of the intervention was to assist patients and families achieve better health with improvements in their eating habits, home food environment, cardiovascular fitness via daily physical activity, and self-efficacy towards health behavior change.

### Medical evaluation and reassessment

The physician performed a complete health history and physical examination. This was important to determine the strengths and challenges facing each family prior to beginning lifestyle change and helps direct an individualized care plan. The physical assessment included evaluation of the patient’s blood pressure, height, weight, BMI Z-score, and growth. All facets of the medical evaluation are in accordance with evidence-based practices for the assessment of childhood obesity [2,35-43].

### Anthropometric and body composition assessment

Height was measured on a wall-mounted stadiometer to the nearest 0.1 (cm). Weight was measured on a calibrated beam balance platform and/or electronic digital platform scale to the nearest 0.1 (kg). Body mass index (BMI) was calculated from height and weight measurements. BMI z-score (BMI-z) was determined using the validated formula [Z={[(BMIcalc÷M)ˆL ]-1} ÷(L×S)] for age and sex, including the parameters L, M and S for non-Gaussian distributions [4]. Criterion body fat was measured by dual energy x-ray absorptiometry (DXA). Whole body scans were performed with a Norland XR-36 bone densitometer (Norland Corporation, Ft. Atkinson, Wisconsin USA) with subject in supine position by the same investigator. Tissue masses were analyzed using software version 3.7.4/2.1.0. The XR-36 x-ray tube operates at 100 kV and uses dynamic samarium filtration (K-edge at 46.8 keV) to produce energy peaks at maximum of 40 and 80 keV. The XR-36 uses dynamic filtration to minimize beam hardening. Dual NaI detectors measure the attenuated x-ray using a pixel size of 6.5 × 13.0 mm and a scan speed of 260 mm/sec. Whole body DXA scans were performed after subjects removed metal objects or clothing containing metal components. Each scan session was preceded by a calibration routine using multiple quality control phantoms that simulate soft tissue and bone per manufacturer guidelines.

### Nutrition assessment, education and counseling

The team dietitians specialize in pediatric weight management, exercise science and motivational interviewing. Education and counseling are founded on the patient and family’s readiness to change, health status and personal priorities. At the initial visit, the RD completed a nutrition behavior screening and utilized a 24-hour dietary recall to collect key information. The RD also assessed the patient’s home food environment(s), nutrition role model(s), suspect food insecurities, as well as, dietary patterns of the patient’s fellow household members. The RD distributed a 3-day food log to the patient and all family members at the initial visit. Time is reserved at the first follow-up visit to review the food logs and to identify key priorities for behavior change. All subsequent follow-up sessions included careful observation of the nutrition care process for overweight and obese children and adolescents.

### Fitness assessment, education and counseling

The clinical EP assessed the patient and family’s fitness level and fitness personalities. At the initial visit, all patients underwent a sub-maximal cardiovascular fitness (CVF) assessment using a treadmill walking protocol to predict maximal oxygen uptake (VO_2_)according to Nemeth (2009); developed and cross-validated on a sample of overweight and obese children [44-45]. The sample CVF is presented in mL/kg/min and L/min to recognize that improvements in the predicted VO_2_ max when measured as mL/kg/min may be due to improvements in the patient’s weight [44,46-47]. The EP provided an activity log at the first visit to capture the subject’s exercise and movement patterns. The EP provided fitness education and counseling with age-appropriate incentives for accomplished goals at subsequent visits to support improvements in behaviors related to exercise frequency, type, duration, physical activity preferences and screen time.

### Motivational interviewing and behavior change

All investigators engaged in motivational interviewing, shared-decision making and sensitive conversations with subjects and their families. The clinic’s philosophy is that a subject’s weight is a product of their habits and thus education and counseling are focused on improving habits that are important to the patient and family. Therefore, few conversations focus on a patient’s weight and changes in weight [36-41].

### Data collection

Data were collected for subjects who completed the 12-month intervention between the years of 2012 to 2015. The University of Wisconsin Human Subjects Committee (Institutional Review Board) approved all procedures. Follow-up visits at 6 and 12 months were defined based on a +/− 3-month window. If there were multiple visits within the +/− 3 month window, then averaged outcome values across the multiple visits were calculated and used for the analyses.

### Statistical analysis

Body composition, CVF, and anthropometric measurements were summarized by standard descriptive statistics using means ± standard deviations. A linear mixed effects model with subject specific random effects to account for correlations between repeated measures was used to evaluate changes in anthropometric, CVF, and BC measures from the baseline assessment. Changes from baseline to the 6 and 12 month assessments were evaluated using a paired t-test. Normal probability plots and the Shapiro-Wilk test were used to examine the normality assumption. If there was indication that an outcome measure was not normally distributed, then a power transformation was applied before conducting the comparisons. All reported p-values were two-sided and p<0.05 was used to determine statistical significance. Statistical analyses were performed with SAS software (version 9.3; SAS Institute, Cary, NC).

## Results

**Figure 1:**
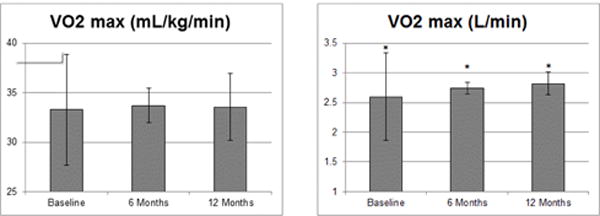
Significant improvements in cardiovascular fitness (CVF) over patient course in a pediatric fitness clinic in patients who completed one year of clinical programming. Mean values displayed with standard deviation (SD) bars. Significance p < 0.05 indicated by asterisk (*).

**Figure 2:**
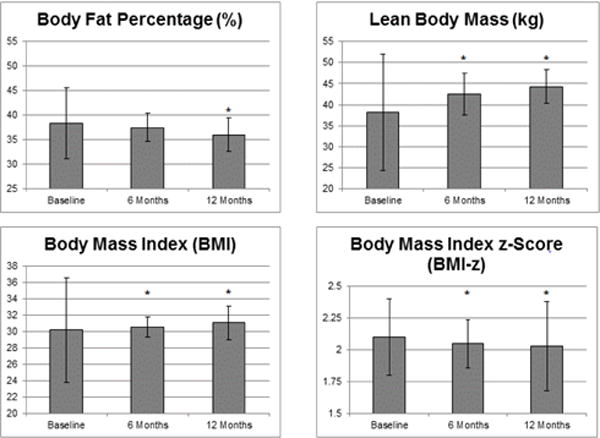
Significant improvements in body composition as compared to body mass index (BMI) over patient course in a pediatric fitness clinic in patients who completed one year of clinical programming. Mean values displayed with standard deviation (SD) bars. Significance p<0.05 indicated by asterisk (*).

The results are presented in Tables 1–2 and Figures 1–2. The baseline demographic, and anthropometric and fitness measurements are presented in Table 1.

Six-month and one year changes in body weight, BMI, BMI Z-score, predicted VO_2_ max in mL/kg/min (standardized for body weight) and L/min (absolute score), body fat percentage and lean body mass are presented as mean ± SD in Table 2.

P-values are provided to indicate statistical significance (p<0.05). Statistically significant improvements were seen at 6 months for BMI-z score (−0.05 ± 0.13, p=0.0108), lean mass (+4.24 kg ± 5.0, p<0.0001) and predicted VO_2_ max (+0.14L/min ± 0.10, p<0.0001). These improvements were sustained and continued at one year for BMI-z score (0.06 ± 0.18, p=0.003), lean mass (+6.0 kg ± 4.0, p<0.0001) and predicted VO_2_ max (+0.22 L/min ± 0.19, p<0.0001). Additionally, significant improvements in body fat percentage were observed at the one-year follow-up (−2.28 ± 3.49, p<0.0001).

Figures 1 and 2 illustrate the mean ± SD values with significance at p<0.05 (indicated by an asterisk). These statistically significant changes in BC and CVF occurred despite significant decreases in BMI-z at 6- and 12-months (2.05+0.3, p=0.007 and 2.03+0.35, p=0.003), increases in BMI at 6- and 12-months (0.37 ± 1.121 p=0.047 and 0.91 ± 2.06, p<0.001) and increasing weight measurements at 6- and 12-months, respectively (+6.60 ± 6.93, p<0.0001 and +6.30 ± 5.80, p<0.0001) (Figure 2).

These data support the limitations of monitoring weight and BMI alone and highlight the utility of other measures to accurately capture and track improvements in BC and CVF.

## Discussion

These data show sustained physiological improvements for 12 months in pediatric patients who successfully completed an evidence-based, multidisciplinary weight management intervention [2,26-34]. Improvements were observed in body composition (increases in lean body mass and decreases in percent body fat), decreases in BMI-z score, and increases in cardiovascular fitness (CVF) over the one year clinical intervention study.

At present, there is an absence of long-term outcomes in the literature for similar measurements of CVF, specifically predicted VO_2_ max, and body composition using DXA. Most studies present outcomes based on changes in the patient’s weight, BMI and BMI z score [35,48-49]. Epstein (2007) describes outcomes from a similar clinical model for study samples with childhood obesity [50]. Improvements in Epstein’s analysis for younger children were stronger than those that were observed in the present analysis (6 months BMI z score values were: −1.46 versus −0.02 and at 12 months: −1.08 versus −0.05). This discrepancy in improvement may be due to the frequency of follow-up within the Epstein programming. The outcomes for this intervention are more similar to findings from the Strong4Life Obesity Treatment Program who showed changes in BMI z-score values of −0.1, at 6–12 months, and −0.2, at > 12 months, and thus BMI stabilization [51].

This study also demonstrates the importance of using valid and reliable methods to evaluate improvements in the subject’s maximal oxygen uptake (predicted VO_2_) and BC, aside from the commonly presented change in a subject’s BMI. Nemet (2005) found that children 6 to 16 years who underwent a weight management intervention had improvements in body fat percentage at 3-months and 12-months (−3.3% and −5.9%) in a small controlled study setting [38]. However, that study used skinfold assessment which has limitations for assessment of BC in an overweight and obese population. Outcomes of the Nemet intervention are attributed to the greater frequency of program visits; six nutrition visits in 3 months versus one to two clinic visits in a 3-month period. This supports the benefits of more frequent encounters with a multidisciplinary team and the value of structured exercise programs.

Consistent with the Nemet [38] approach, this study utilized frequent encounters, a multidisciplinary team and structured exercise programs. The goal was to measure the clinical outcomes at baseline, 6-months and 12-months, however some subjects were unable to complete the mid-point evaluation (between the allotted time-frame of three to nine months) and this accounts for our sample size difference at 6-months.

We attribute the sustained changes in our study to three facets of the clinical intervention: (1) evidence-based clinic procedures using valid and reliable methods for assessing change, including assessment of BC and CVF, (2) a multidisciplinary approach that utilized specialized allied healthcare providers at regular intervals, and (3) individualized patient and family-centered care with reliance on motivational interviewing techniques in education, counseling, and goal-setting. The literature supports that healthcare practitioners who utilize facets of motivational interviewing may be well positioned to engage in heath behavior change talk with patients and families [36-41]. A clinician’s language, tone, and intention can empower or debilitate a patient and family from making critical health behavior changes [52-55].

This study illustrates that application of evidence-based weight management protocols can produce sustainable health outcomes for overweight and obese youth in a clinical setting [2, 32-34, 40-43]. This study also provides evidence that BMI alone may not represent an individual’s progress towards improved health as it does not accurately describe CVF and is not a reliable indicator for significant changes in BC in overweight and obese children and adolescents [43]. These data demonstrate changes in BC and CVF that would have been missed with the use of BMI and weight changes alone.

## Limitations

Limitations of the present study include attrition and the assumption that the most complex and most ill patients are often those with the longest care plans. Attrition rates in pediatric obesity clinics have been shown to vary from 49–73% [56]. The variables explored that correlated with patient attrition are consistent with those in the literature and include non-Hispanic black ethnicity, mental health issues, overall health status, difficulty with medical insurance coverage, location and timing of program visits, unfulfilled parental expectations, and a child’s desire to leave the program [57-60]. Additionally, we assume that the most complex and/or ill patients require the longest care plans. This skews clinical outcomes since patients who progress more quickly than their counter-parts conclude their clinic course earlier; these patients have more positive short-term changes and thus the clinic does not have long-term follow-up data. This scenario leaves patients who struggle with behavior change but remain dedicated to the program and have more poor outcomes. Additional limitations are that most patients are referred fairly “late” in the process of weight gain/obesity, as most of the patients had a BMI >99th percentile at baseline. Treatment may be more effective if patients are referred when they are at BMI-for-age percentiles that indicate an increase in risk for overweight (near the 85th percentile) or overweight (85th to 95th percentiles) [2, 28-31, 36-37].

Primary care clinics and patient’s families and caregivers play an important role in the screening and treatment of overweight and obesity. These data support that there is a need for greater collaboration among primary care clinics and specialized weight management programs in the prevention of obesity and treatment of overweight and obesity [26-32,61]. Present research supports the need to refer at-risk for overweight and overweight children and adolescents to higher level treatment earlier.

## Conclusion

Interventions are frequently evaluated on basic measures such as changes in the subjects’ weight, skinfolds, BMI and BMI z score, and for short duration [35,38,48-49]. However, in the present study the criterion measures to evaluate change included 12 month changes in whole body DXA scans to track subject fat mass, lean body mass and percent body fat and a population-specific treadmill test to evaluate changes in cardiovascular fitness and predicted VO_2_ max [44-45]. Based on the aforementioned measures, this study indicated that statistically and clinically significant improvements in cardiovascular fitness and body composition could be achieved and sustained over a one year period in a sample of 79 overweight and obese children via an individualized, multidisciplinary clinical intervention.

## Figures and Tables

**Table 1:** Baseline demographic and clinical characteristics of study sample captured at baseline.

Parameter	Patients Who Completed One Year N (%)	P-Value^†^
Gender		
Female	41 (52)	0.832
Male	38 (48)	
Age (Mean +/− SD)	11.1 ± 3.3	0.1136
Preferred Spoken Language		0.001
English	59 (76)	
Non-English	19 (24)	
Anthropometric and Fitness Measures (Mean +/− SD)		
Body Mass Index (BMI)	30.2 ± 6.4	0.9034
BMI-Z score	2.1 ± 0.3	0.0262
Weight (kg)	71.3 ± 28.4	0.504
Fitness (VO_2_ max, mL/kg/min)	33.3 ± 5.6	0.8765
Fitness (VO_2_ max, L/min)	2.6 ± 0.7	0.046
Body Fat Percentage (%)	38.3 ± 7.2	0.9477
Lean Body Mass (kg)	38.2 ± 13.8	0.5035

± Based on generalized estimation equation (GEE) analysis

**Table 2:** Mean (SD) absolute changes in anthropometric and fitness measures from baseline to month 6 and month 12 months among all study.

Parameter	6 Months				12 Months			
	N	Mean	SD	p-Value‡	N	Mean	SD	p-Value‡
Body Mass Index (BMI)	45	0.37	1.21	0.047	79	0.91	2.06	0.0002
BMI-Z score	45	−0.05	0.13	0.0108	79	−0.06	0.18	0.0025
Weight (kg)	45	6.6	6.93	<0.0001	79	6.3	5.8	<0.0001
Fitness (VO_2_ max, mL/kg/min)	29	0.41	1.77	0.2203	71	0.26	3.37	0.5202
Fitness (VO_2_ max, L/min)	29	0.14	0.1	<0.0001	71	0.22	0.19	<0.0001
Body Fat Percentage (%)	29	−0.82	2.89	0.1387	79	−2.28	3.49	<0.0001
Lean Body Mass (kg)	29	4.24	5	<0.0001	79	6.02	4	<0.0001

‡ p-Value for evaluating change from baseline (initial visit, initial assessment)
